# RFID-Based Crack Detection of Ultra High-Performance Concrete Retrofitted Beams

**DOI:** 10.3390/s19071573

**Published:** 2019-04-01

**Authors:** Benjamin Bruciati, Shinae Jang, Pierre Fils

**Affiliations:** Department of Civil Engineering, University of Connecticut, Storrs, CT 06269, USA; benjamin.bruciati@uconn.edu (B.B.); pierre.fils@uconn.edu (P.F.)

**Keywords:** radio frequency identification, structural health monitoring, ultra-high-performance concrete, damage index

## Abstract

Ultra-high-performance concrete (UHPC) is a novel material with multiple known uses and many still yet to be discovered. Recently, the use of encasing welded shear studs in UHPC on the web of corroded steel beams was developed. This creates a bearing force transfer mechanism to bypass the corroded web plate. This new material and its uses come with many uncertainties in the short and long term. Structural health monitoring (SHM) can be a tool to observe the development. Specifically, radio frequency technology (RFID) can be used. RFID has existed commercially since the 1960s and has been used as a crack sensor before, but never with UHPC. RFID-based crack sensing is being used to monitor the UHPC retrofit. A crack is simulated on the UHPC specimen and then a commercial, low cost tag is secured. Using backscatter power, the tag reads the crack existence and its increasing volume with every new damage stage. Using a damage index, comparing the uncracked and each cracked stage, this method is not restricted to the raw received signal strength indicator (RSSI), which could be different at each tag. With this sensor, the small cracks that occur in UHPC during its creation can be monitored to ensure the capacity of the retrofitting is maintained. The tested RFID-based crack sensor can be used on various other forms of UHPC.

## 1. Introduction

The main goal of civil engineers is to design, build, and maintain infrastructure. The continued growth of our society is reliant on the transportation network and dependent on its integrity. This growth along with the aging infrastructure puts the integrity at risk. According to the 2017 Infrastructure Report Card [1], the U.S. received a D+ for the overall infrastructure and a C+ for bridges [1]. In fact, 9.1% of bridges are structurally deficient but are traveled over about 188 million times per day. Coupled with the increasing traffic loads and natural disaster impacts, bridges pose a major safety risk for the nation.

In these structures, concrete is one of the most widely used building materials worldwide [2]. The first use of iron-reinforced concrete was by the French builder François Coignet in Paris in the 1850s [3]; ever since then, reinforced concrete has been a staple of modern construction. However, because of its poor tensile resistance and cracking behavior, researchers hare intent on developing a new cementitious material that could replace or strengthen the reinforced concrete for infrastructure.

Ultra-high-performance concrete (UHPC) is a relatively new division of metal reinforced concrete that, in its present form, became commercially available in the United States in about the year 2000 [4]. The Federal Highway Administration (FHWA) began investigating the use of UHPC for highway infrastructure in 2001 and has been working with the transportation departments of the states to deploy the technology since 2002 [5]. Currently, the most popular application for UHPC in prefabricated bridge construction in the U.S. is in the connections between prefabricated bridge deck elements. With the increasing popularity of using UHPC for building columns, bridge girders, for structural repair, etc., more uses for it are being found [6]. Its material properties make it a more feasible material for bridge connection joints and structural repair. The introduction and increasing use of this new material has come with new maintenance and structural health monitoring (SHM) practices. Using SHM, these new applications for UHPC can be evaluated.

An estimated $123 billion were spent on bridge repairs in 2017 [1]. With structural health monitoring (SHM), this estimate can be decreased. This tool can evaluate the health and safety of any engineered structure during normal and extreme excitation. SHM can re-evaluate structures in real time and identify what needs to be further investigated. Manual inspections, while primarily visual, are unreliable and sometimes impossible depending on the nature of the damage and the access [7]. Many non-destructive techniques such as ultrasonic and eddy current pulsed thermography (ECPT) were developed to have acceptable resolution and reliability. However, these methods are costly and were not designed for large scale use. Low cost wireless sensors became the most attractive solution to solve the restrictions of the previous methods.

Originally designed for large scale asset tracking [7] radio frequency identification (RFID) technology, can fill this new role in SHM due to its unique capabilities. Ultra-high frequency (UHF) passive RFID tags are wireless, low cost, and easily deployable in high numbers. These tags have the capability to track the permanent change in impedance and radiation caused by a crack. Therefore, when a material under and around the tag changes, the loss of that material can be monitored. This capability makes RFID a viable crack sensor.

The maintenance of aging infrastructure is critical to achieve the designed structure life. Corrosion damage affects 15% of the nation’s bridges [1] and is severely shortening their service life. This damage is particularly severe at the girder ends. This is where water and deicing materials leak through the joints and create section loss at the ends. This section loss reduces the thickness of the web plate near the supports and affects the baring capacity of these girders [8]. The current repair procedure is time consuming and costly. New methods, proposed by Kennedy and others, look to use UHPC as a retrofitting. With Kennedy’s UHPC jackets having only been implemented in June 2014 [9], this new repair method needs to be studied in the field long term. With much focus on numerical simulation, for the future use of UHPC, by researchers like Huang [10] and Liu [11], the already in-place structures lack the required attention. Current retrofits like Kennedy’s increase the section size, which can cause unknown forces or transfer previous ones. These retrofits are missing the SHM technology required for maximizing maintenance procedures in terms of time and these new forces. A traditional monitoring method would be via electrical resistance strain gages. This method, while accurate, is costlier and more prone to damage during and after installation [12].

A cheaper method would be RFID-based tag crack sensors. Previous research of crack sensors by Kalansuriya [13] and Martinez-Castro [14], while successful on both concrete and metallic materials, respectively, lacks the validation of testing with UHPC. This system used passive RFID technology but with key modifications, such as the removal of the substrate material and the use of a volume-based index rather than just crack width and length like many other inspection procedures. So far, a system with low cost, field ready applicability and testing does not exist. The ease of large-scale deployment, the low cost and the inspection feasibility of the 1 ft (30.48 cm) read distance make this an ideal tool for transportation agencies to monitor these new UHPC retrofits. Other previous research, by Zhang et al., outlines a system that can assess the condition of a structure through the use of RFID tags. This system, while having been successfully tested on PVC tubes, also lacks testing on UHPC. The system also requires a more complex strain sensor setup and unique conditions such as conductive paint and the placement of a material referred to as a brittle bar [15]. The ease of placing a sticker-like tag that could be seen by a technician and the ability to place it anywhere on UHPC section without any major surface changes, like painting, could increase the range of locations where this system could be implemented.

This paper shows the applicability of RFID tags as crack sensors for UHPC. The cracks were located on UHPC specimen sections. The crack was simulated by saw cut and increased in volume at each damage stage. The damage sensitivity was determined by the ability of the tag to return different backscatter powers as the crack volume in the UHPC specimen changed. The backscatter power was then measured by two different methods in lab scale experiments. The system was able to perform the crack monitoring even with adverse effects from the UHPC. The total damage index showed the change in backscatter power and was used to quantify the results.

## 2. Theoretical Background

The theoretical background behind the crack sensor is summarized in this section. The use of backscatter power for this passive RFID tag was based on the wave equation. The RSSI, could then be obtained and used later, as a measured value, for crack detection. The RSSI was then compared to a new damage index that related to the volume of the crack. Using the shape of a right triangular prism, the crack was idealized and able to be quantified into a new volume-based damage index. With the new damage index and RSSI, comparison of the two was done with the total damage index. This related the RSSI of the undamaged and the damaged state. The form of the total damage index could bypass the variability issue of the RFID tags by using only its own RSSI values as the comparison. This damage index can then be related to the volume index for a means of crack quantification.

### 2.1. Backscatter Power

The antenna-based crack sensor is based on the idea that when a crack appears, it permanently changes the radiation and impedance characteristics of the antenna [13]. In this research, the ultra-high frequency (UHF) passive RFID tag crack sensor uses this idea and treats the backscatter power as the crack measurement unit. Passive refers to the tag either harvesting or receiving power from a power read-out unit during data collection [16]. Backscatter power is identified as the most important measured value for crack detection [13]. This power comes from the transmitted power of the antenna which is then transferred to the tag. The tag then sends the power back to the antenna with a loss corresponding to the tag’s impedance and radiation change, which is affected by the crack under the tag. This difference between the transmitting and received powers is the backscatter power. Using the wave equation, backscatter power can be described as follows:

(1) $$P_{R}=\frac{P_{T}\left(G_{T}G_{R}\right)}{4\pi }{\left(\frac{\lambda }{{\left(4\pi R_{T}R_{R}\right)}^{2}}\right)}^{2}\sigma$$

where *P_R_* is the backscatter power (dB), *P_T_* is the transmitted power (dB), *G_T_* is the transmitting antenna gain (dB), *G_R_* is the receiving antenna gain (dB), *λ* is the signal wavelength (m), *R_T_* is the distance between the target (the RFID tag) and the transmitting antenna (m), *R_R_* is the distance between the target and the receiving antenna (m), and *σ* is the targets radar cross section (m^2^). If the transmitting antenna and the receiving antenna are the same, *R_T_* = *R_R_*. Since the backscatter power is in dB, and dB are unit-less, the backscatter power is a logarithmic way of describing the ratio between the power sent and received. Equation (1) shows that reading distance affects backscatter power. As read distance increases by a quartic factor, the power is decreased. *P_T_* can be determined using the received signal strength indicator (RSSI) logged by the reader equipment from the following equation. *P_R_* can be determined using the RSSI logged by the reader equipment from the following equation:

(2) $$\mathrm{RSSI}=10\log_{10}\left(\frac{P_{R}}{1\mathrm{mW}}\right)$$

where RSSI, which is in dBm (decibel milliwatts), is an expression of backscatter power. Most reading equipment uses a spectrum of reading frequencies. This changes the value of RSSI depending on the transmitting frequency channel. In North America, the signal frequency varies in the range of 902–928 MHz. The Impnj Speedway MultiReader software performs a hopping frequency data collection sequence in this range of frequencies during each collecting session. Through a MATLAB script, the gathered RSSI values can be averaged and the averages can be equally weighted across all frequency channels.

### 2.2. Volume Index

**Figure 1:**
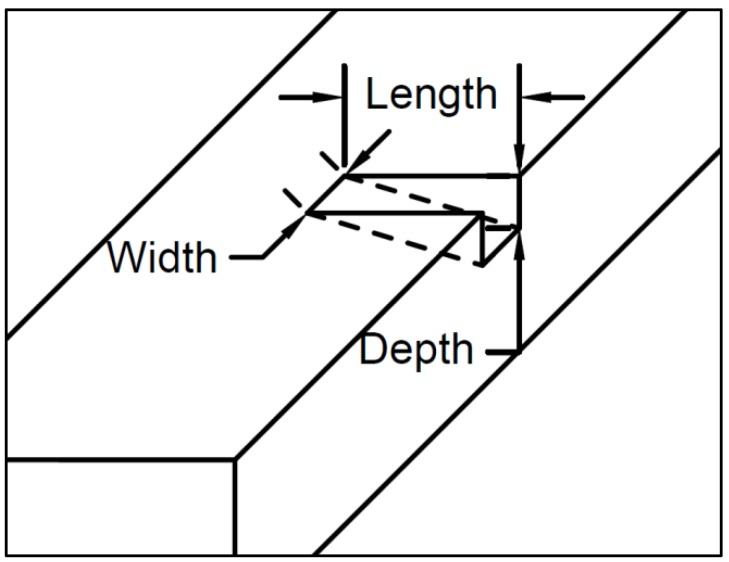
Crack dimensions.

UHPC is a heterogeneous, cementitious composite material, with discontinuous fiber reinforcement, and a discontinuous pore structure [5]. With these voids and reinforcements present, almost at random, the inner structure of this material can be less predictable than RC. Voids can appear within the first mm of the surface and the same goes for the reinforcement. For more realistic damage detection, a new index based on loss of volume was used. Usually, for visual inspection, especially for RC, cracks are measured in length and width [17]. However, to get a more complete idea of the crack, we also measured the depth. Using a right triangular prism shape, the crack was idealized and able to be easily measured. Using the length, width, and depth of the crack, the volume index was obtained. This is shown in Figure 1.

With this right triangular prism shape, the crack was idealized and not subject to the disorder of the material. This may not account for the voids that can appear in this material, but this index is superior in that it describes the three-dimensional nature of the crack.

### 2.3. Damage Index

Due to the nature of the hopping frequency data collection method, all data that is presented is in terms of mean RSSI. There are many ways to quantify this mean data. Two main ways are the actual value of the RSSI and s comparison of two data states. These two data states would be the intact (RSSIi) state and the damaged state (RSSId). This index changes with each new damage stage. This will be referred to as the total damage index (TDI), shown as a percent. Thus, the total damage index is defined as:

(3) $$\mathrm{TDI}=\frac{\mathrm{RSSIi}-\mathrm{RSSId}}{\mathrm{RSSIi}}\times 100$$

where with the total damage index, crack identification and crack size changes can be correlated. When the total damage index value is a non-zero number, the crack volume has changed. The total damage index can take into account the non-uniformity of the tags. Due to the mass production of the RFID tags, no tag is perfectly identical. This characteristic would invalidate the use of the distinct values of mean RSSI from each tag. Due to this, each tag reading needs to be compared to its own RSSI values by using the total damage index. If the tag is placed over the crack when the crack is in an early stage, it can be monitored. If the total damage index value is non-zero, then the crack volume has changed since tag’s application.

## 3. UHPC

Ultra-high-performance concretes are utilized for applications such as bridges, pavement overlays, and tall building columns [5]. These applications sometimes call for UHPC over normal reinforced concrete (RC) due to its material properties. UHPCs are cementitious composites characterized by high compressive strength, low water binder ratio, and optimized gradation curves [4]. In many cases, thermal activation, fiber reinforcement, and superplasticizers are employed to increase strength, enhance ductility, and ensure workability [18]. Specific characteristics that distinguish UHPC from RC include compressive strength above 21.7 ksi (150 MPa) and pre-and post-cracking tensile strength above 0.72 ksi (5 MPa). Fatigue tests of UHPC specimens under various combinations of stress level and stress range showed a range from 2.5 to more than 7.0 million cycles [5]. At the lowest end of this range, this fatigue life was about 80% more than RC [19]. The fibers contained within the concrete had a large impact on the tensile capacity. The steel fibers improved the flexural moment capacity, stiffness, and cracking behavior of the UHPC beams, but the crack localization caused a reduction in ductility. This phenomenon can occur in both normal and high-strength concretes containing steel fibers and conventional reinforcement [20]. These characteristics show the advantages of UHPC over RC, but at a cost.

UHPCs have a higher monetary cost than RC due to the difference in material properties. The commercially available product is a proprietary blend sold for about $2000/yd^3^ ($2600/m^3^) versus the conventional concrete which is about $100/yd^3^ ($130/m^3^) [21]. Given that UHPC is 20 times more expensive than conventional concrete, it is generally used for retrofitting rather than massive construction. Although it has a higher performance, UHPC has minor drawbacks such as early-age cracking during the manufacturing process, due to the high cement content and highly exothermal hydration reaction [22], and cracking, caused by secondary forces such as temperature and shrinkage loads, during and after the manufacturing process [23]. During a retrofit, a structure can experience unusual forces and, after the retrofit is complete, this new added section can experience unknown forces from section size change or structure material UHPC interactions. These unknown variables cannot always be accounted for; therefore, unintentional cracking can occur during and after retrofitting. Cracking can lead to exposure of the fibers to the elements. This greatly decreases the tensile capacity of the UHPC. Once exposed, cracking would make the retrofitting unreliable if the beam was still exposed to the environment. Luckily, if the metal content and length are increased, shrinkage crack surfaces are considerably reduced, up to 86% compared to non-fiber specimens [24]. With timely crack detection, these cracks can be identified and repaired before significant damage to the inner fibers occurs. Because the location of UHPC-retrofitted spots would be known, the detection of cracks with the low-cost RFID is feasible. This would improve UHPC’s reliability.

## 4. Methodology

The UHPC specimen, sensor development, damage detection procedure, and experiment design are described in this section.

### 4.1. Specimen

**Figure 2:**
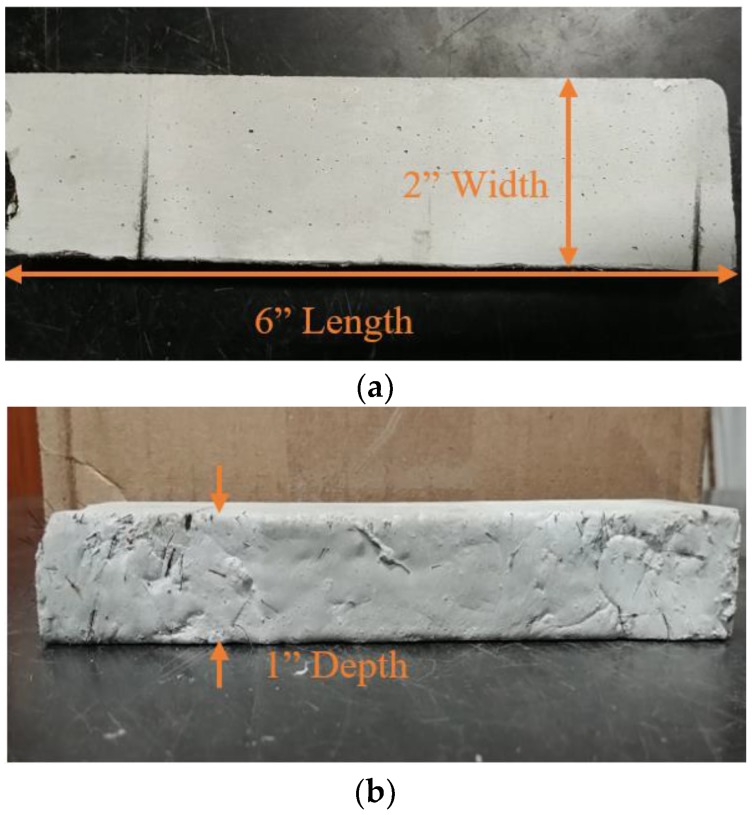
Ultra High Performance Concrete Specimen with metal fibers (UHPC-A). (**a**) Top view; (**b**) Side view.

Two UHPC specimens were fabricated for the experimental validations, UHPC with metal fibers (UHPC-A) and UHPC without metal fibers (UHPC-B). Both specimens have the same constitution and dimensions. The dimensions of the fabricated UHPC specimens are 1 inch (2.54 cm) in depth, 2 inches (5.08 cm) in width, and 6 inches (15.24 cm) in length, as shown in Figure 2. The multiple cracks shown in Figure 2a are from the multiple tests done on each sample for this paper. UHPC-B has multiple cracks as well. The crack dimensions were different to ensure the range of the RFID tag. In Figure 2b, the metal fiber reinforcement is shown protruding from the sample.

The specimen was a common commercial mix Ductal JS1212 (UHPC-A), produced by Lafarge North America [25]. The UHPC formulation contained premix power, water, Premia 150 (a modified phosphonate plasticizer), Optima 100 (a modified polycarboxylate high-range water reducing admixture), Turbocast 650A (a non-chloride accelerator), and steel fibers. The steel fibers included in this mix design were non-deformed, cylindrical, high-tensile strength steel with a diameter of 0.008 in (0.2 mm) with a length of 0.5 in (12.7 mm). The steel tensile strength was specified as greater than 290 ksi (2000 MPa). The steel fibers had a thin brass coating providing lubrication during the drawing process and corrosion resistance for the raw fibers [26]. This metal working process was used for the reduction of the cross-section of the rod [26]. A steel fiber content of 2% by volume was used for UHPC-A. The composition of UHPC-A, by weight, is shown in Table 1.

### 4.2. Damage Description

For the laboratory-scale validation, crack damage was simulated as a thin cut. The cut was made with a saw with a blade thickness of 0.03 inches (0.762 mm). Hasgul et al. [20], while studying failure modes, deflection/curvature ductility, and cracking patterns, used four-point bending tests to induce cracks in the UHPC. At the maximum strain, their UHPC experienced crack widths of 0.15 inches (3.8 mm). In this paper, the crack width was simulated up to 0.08 inches (1.9 mm) in the first step. This width is similar to fatigue cracks shown after 20,000 cycles [15], or cracks from forces such as temperature or shrinkage [22]. The decision to attempt to match the smaller cracks mentioned previously was due to the likelihood of a crack of that size occurring. After 20,000 cycles, temperature or shrinkage cracks are much more likely to occur than the maximum strain cracks created by Hasgul, due to the extreme circumstances that need to occur for maximum strain. The saw cut was very carefully made straight; however, the cut section could have chips and voids due to the intrinsic characteristics of the cementitious material. Saw cuts were made as uniform as possible, however, the crack volumes for each test could vary. This crack was simplified, as a representative of any cracks caused by bending, fatigue, or axial loads. The crack dimension was measured using a dial caliper that measures up to 10,000 thousandths of an inch from zero to six inches (0 to 15.24 cm).

### 4.3. RFID-Based Crack Sensors with Modifications for UHPC

**Figure 3:**
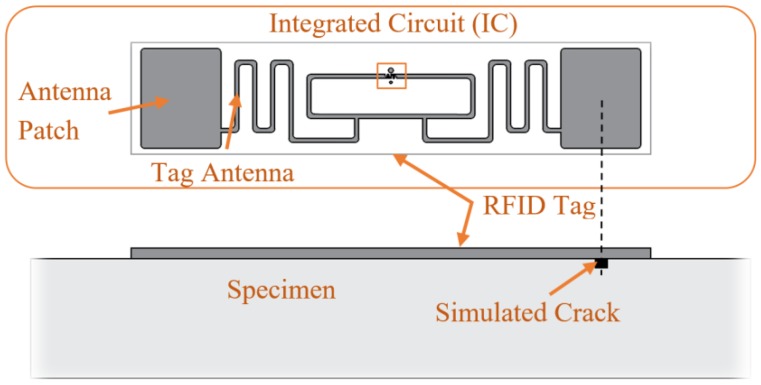
Commercial Radio Frequency Identification Tag.

The commercial UHF RFID tag used in this research was the Alien Technology ALN-9662 Short Inlay tag [27], as shown in Figure 3. This tag is EPG Gen 2 and ISO/IEC 18000-6C compliant and it uses a Higgs 3 EPC Class 1 Gen2 RFID tag integrated circuit (IC). The tag antenna is made of a flexible metallic material, which is adhered to a wet inlay. The cost for each dipole tag is about $0.10 for mass production. The antenna sensors can be fabricated on inexpensive substrate materials, such as paper, PVC (polyvinyl chloride), using low-cost fabrication techniques, such as inkjet printing [7]. This commercial tag is 70 mm long and uses a Higgs 3 EPC Class 1 Gen2 RFID tag integrated circuit (IC). Figure 3 shows a detailed view of the IC and the tag mounted on the specimen. As shown, the antenna patch is oriented across the crack for test replicability and crack sensitivity uniformity. While this testing places the patch directly over the crack for test replicability and to ensure best possible crack sensitivity, the tag can still sense cracks not in that location as proved by Martinez-Castro. In addition, different than the previous study of crack detection on metallic surfaces by Martinez-Castro et al. [28], the substrate between the tag and the specimen was removed. Therefore, direct tag attachment is feasible for crack detection on cementitious materials.

Because RSSI is affected by environmental factors near the sensor, sensor attachment methods should be identical at the different damage stages. The patch part of the tag was placed over the crack. The sensors were applied using 1 in × 0.25 in pieces of scotch tape, one on each corner. This secured the tag to the face of the specimen. The same application procedure was used for each tag, specimen and tested to ensure no other variables, besides the crack size, changed between each test. The attachment method was carefully tested via separate preliminary tests to control all the variables. This tag securing method is for trial uniformity, not to ensure the capture of the extension of the concrete. Other factors such as radio wave reflection by the steel fibers and wall structures were kept constant. The testing was performed in a lab where these factors were able to be kept constant, as was the two system identical reading setup/location for steel fiber effect minimization. In practice, this uniformity may not be possible if the structure has undergone repair since the tag was applied. The detailed logistics on sensor operation and replacement for field implementation is recommended. As the total damage index, which compares different damage states all with the same tag application, was used in all tests, the tape and other environmental changes had no effect on the results if kept constant.

### 4.4. Measurement Setup

**Figure 4:**
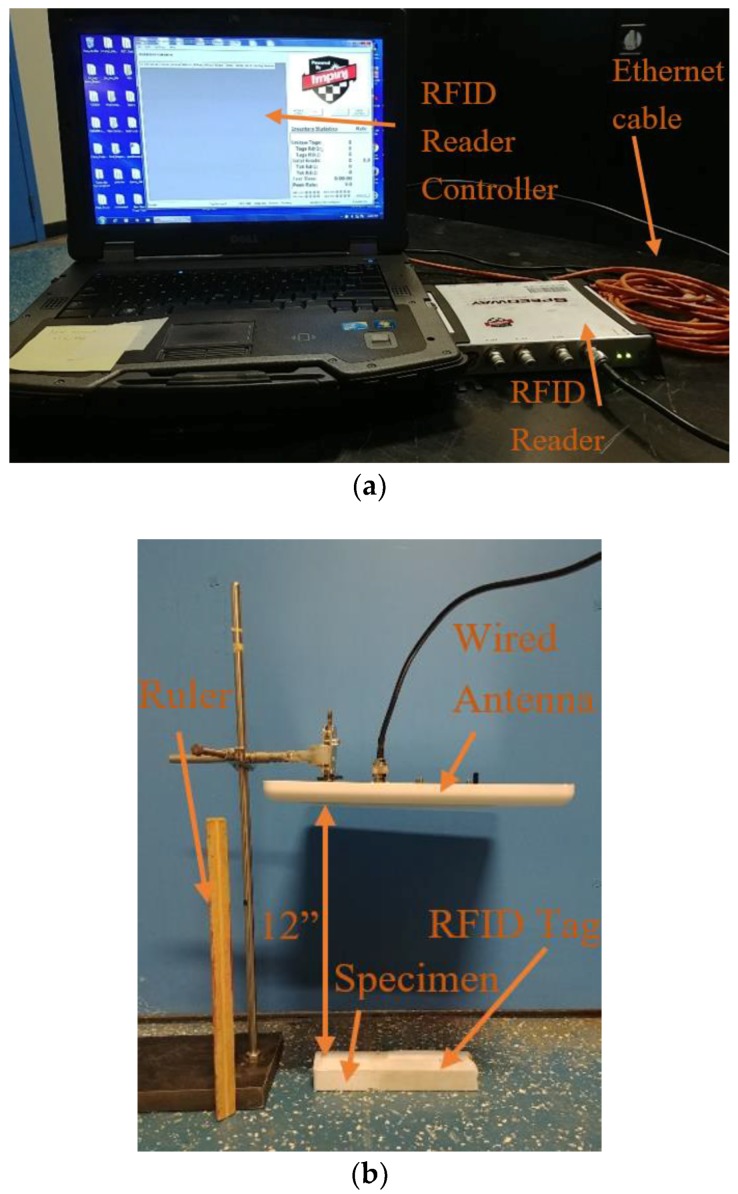
Lab-based system crack detection setup, (**a**) RFID controller and reader; (**b**) RFID specimen, tag and antenna.

The reflected power from the RFID tag was measured with two different systems, a lab-based system and a handheld system. The lab-based system consisted of a PC with Impnj MultiReader software connected to an Impnj Speedway Revolution R420 UHF RFID reader, shown in Figure 4a. A high gain circular right-hand polarized patch antenna was connected to the reader. Figure 4b shows the lab-based system setup at a read distance of 12 inches (88.9 cm).

In a previous test the read distance of 35 inches (88.9 cm) was used. This was only for one test to confirm preliminary read-distance tests from the previous publication [14]. The previous publication also confirmed the accuracy and usability of this setup. The setup was slightly varied for the other, smaller, read distances.

For data collection for the lab-based system, the MATLAB file averaged all the RSSI values from all the frequencies used by the antenna during the channel hopping sequence. It then displayed the mean RSSI value used in this study, as well as the unique identification for each tag. The lab-based system showed consistent damage identification performance in the past, however, it was lacking mobility for field implementation.

**Figure 5:**
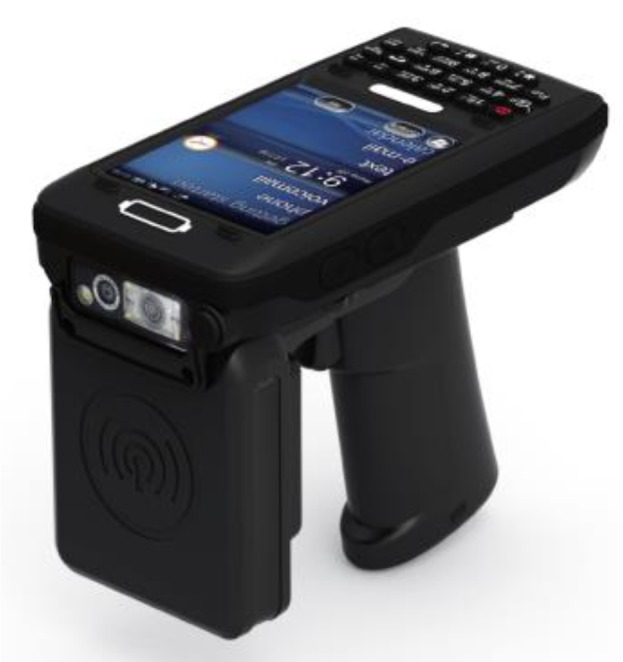
Handheld system.

To improve mobility for field damage identification, another RFID reader with handheld functionality, i.e., ‘handheld system’, was employed. The handheld system used ATID AT870N PC equipped with a windows embedded mobile (see Figure 5). This PC had a UHF module read/write RFID tag with a performance frequency of CE 850–968 MHz; 902–928 MHz are the frequencies used for North America. Using the Alien Handheld RFID reader app, the mean RSSI could be determined by a 1 Hz visual recording method, as the reader did not output a data file. The collected RSSI measurements were then averaged to create the mean RSSI. This system’s applicability was also tested as it was not tested in previous research [14]. With acceptable performance that could overcome the difficulty in maintaining constant read distances, this system could be implemented in the field. This method will be referred to as the “handheld system” because of its mobility and field applicability. When compared to the lab-based system, the handheld’s ease of use is much higher. Without the need of any cables or a power source, because of being battery powered, if fully implemented, this crack monitoring system using the handheld reader would be superior.

**Figure 6:**
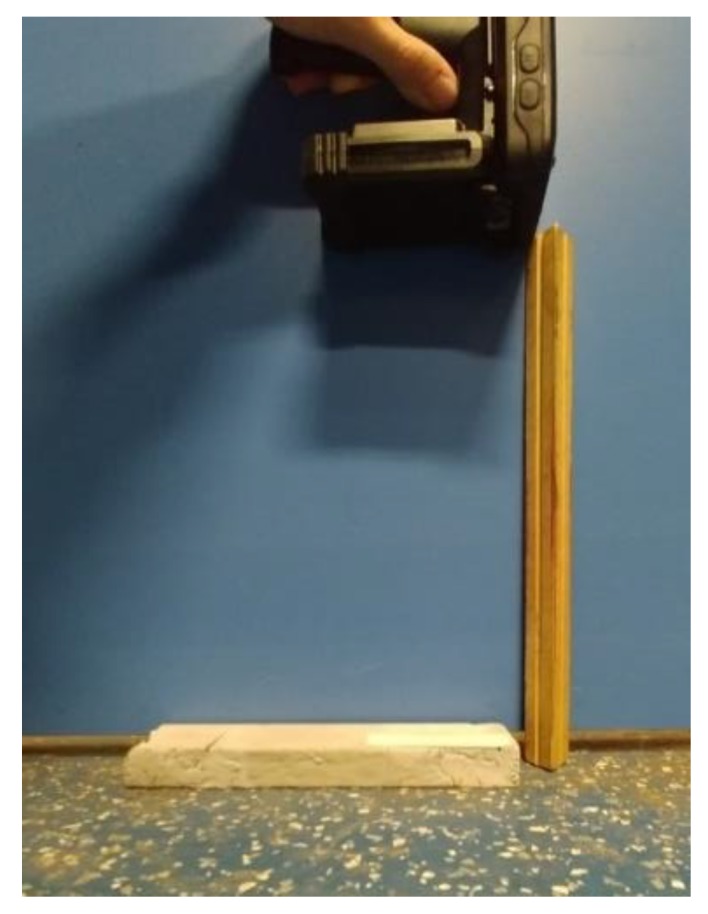
Handheld system crack detection setup.

The front attachment of the mobile PC is the UHF reader, shown in Figure 5. When the search for the RFID signal was initiated, the mean RSSI appeared on the screen along with the tag’s unique ID. Figure 6 shows the data collection setup for the handheld system with a read distance of 1 foot, which will be used for all handheld collection.

### 4.5. Experimental Validation

To validate the performance of the developed RFID-based crack sensors, a series of laboratory-scale tests were conducted.

#### 4.5.1. Test 1: Read Distance Identification

To confirm the read distance of the RFID tag on the UHPC specimen, which increased, the antenna read distance of the mean RSSI value needed to be evaluated. Three tests were performed with various setups and readers. In the first test, four different tags and three different read distances per tag were examined. The three distances were 12 inches (30.48 cm), 24 inches (60.96 cm), and 35 inches (88.9 cm). These distances were chosen because, in Martinez-Castro’s work, the distances were every 3 feet (91.44 cm). Therefore, 1 foot (30.48 cm) intervals under three feet were untested. Due to each RFID tag having a unique backscatter power and the non-uniformities in mass production, four tags were used. These tags were then placed in the same location on the specimen for each read distance. The location was in a center vertical and center horizontal position (center–center) on the UHPC-B specimen. UHPC-B was used to minimize the effect of the metal fibers on mean RSSI.

**Figure 7:**
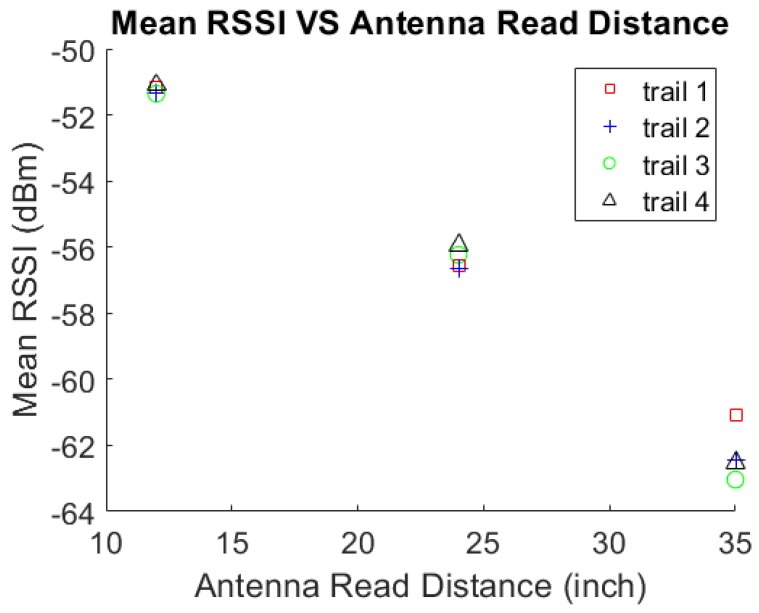
Mean RSSI vs. antenna read distance.

Figure 7 displays the results of four different trials. In each trail, the mean RSSI value was read at three different distances. As expected, the value of the RSSI decreased as the read distance increased. Equation (1) confirms the validity of this trend. Due to the quartic power reduction by distance, only one foot intervals were investigated. Above three feet, the handheld system was unable to detect RSSI. Therefore, distances past three feet were not investigated, with one foot having the highest RSSI, this read distance will be used in the further experiments to sense changes in RSSI more clearly in both reading methods. This one foot measurement distance is also feasible for field use. Being able to be within one foot of any location on the bridge is reasonable.

#### 4.5.2. Test 2: Crack Detection Using the Lab-Based System

**Figure 8:**
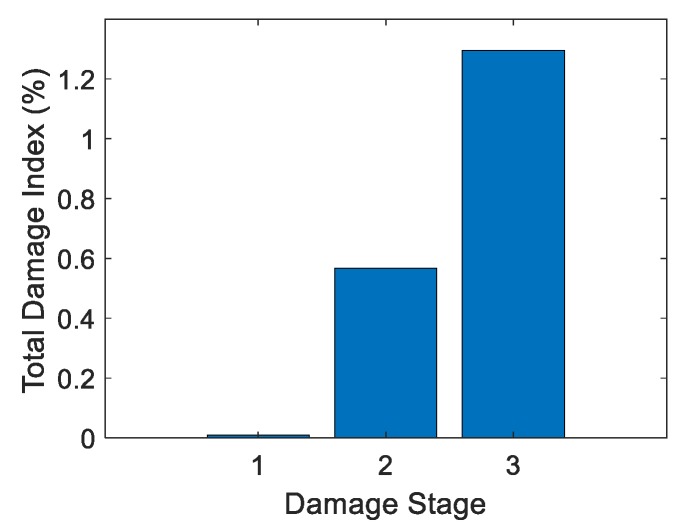
Total damage index vs. damage stage: Test 2a.

The goal of Test 2 was to detect changes in mean RSSI with increased damage. Test 2 used only the lab-based system and tested UHPC-A and UHPC-B specimens. Test 2a used just UHPC-A with the lab-based system with three damage stages and the undamaged stage. Table 2 shows the increase in crack volume with respect to each damage stage and the total damage index. Figure 8 shows the corresponding graph. Studying the table, the trend of total damage increased with increased crack volume.

**Figure 9:**
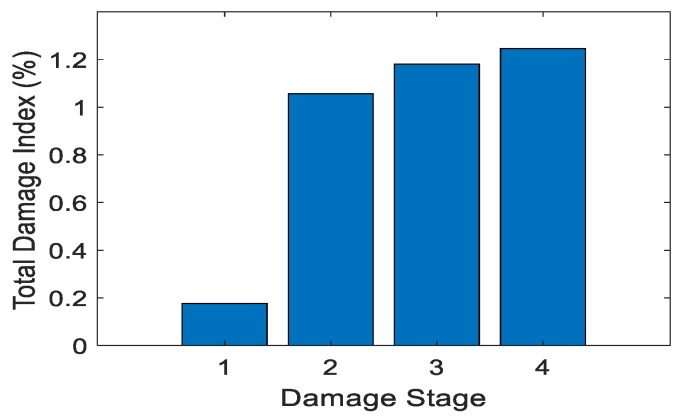
Total damage index vs. damage stage: Test 2b.

Test 3 was crack detection using the lab-based system for UHPC-B, the specimen without metal fibers. The non-metallic reinforcement was the only thing that was different from the Test 2a. Again, the crack was simulated with different volumes with each damage stage. This test was to determine if the fibers heavily influenced the system. Figure 9 and Table 3 show the results of Test 2b.

Test 2b also yielded a positive trend of RSSI using the damage index. Test 2b showed that with the increase in the damage stage, which was an increase in crack volume, an increase of total damage index occurred.

The tag had a positive trend with the largest increase being in Stages 1 and 2. This is believed to be because the crack volume was so small in Stage 1 as to be almost unrecognizable. The crack became significant in Stage 2; there was a large change in the radiation, which then increased the damage index. The change between damage stages may not have been as large in this test as in the previous but the trend still follows. Test 2a,b identified the effect of the metal fibers on the system. As predicted the metal fibers reflected more of the signal, as shown when comparing Stage 3 in Test 2a and Stages 3 and 4 in Test 2b. In Test 2b, Stages 3 and 4 had a higher crack volume than that of Stage 3 in Test 2a. Even with this higher volume, Stages 3 and 4 in Test 2b, which were without the metal fibers, had a lower total damage index than that of Stage 3 in Test 2a. The difference in the two tests was noticeable but not overly important as long as the crack could still be detected with or without the fibers.

#### 4.5.3. Test 3: Crack Detection Using the Handheld System

**Figure 10:**
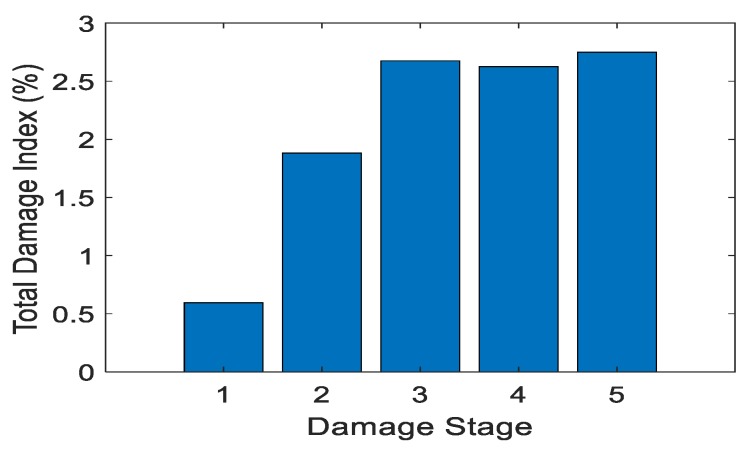
Total damage index vs. damage stage: Test 3.

Test 3 examined the performance of the handheld RFID crack detection system on UHPC-B, the specimen without metal fibers. The handheld system was used in the field as the inspector’s tool for monitoring these cracks. In this test it was critical that the system identify the crack and its increasing volume. Table 4 shows the increase in crack volume with respect to damage stage and total damage index. Figure 10 displays the corresponding graph.

Figure 10 displays the positive trend of the total damage index while the damage stage increased. This was the same trend that Test 2, with the lab-based system, found. However, the handheld test showed larger values for the total damage index. This could be because the non-uniformity of the UHPC material affected the readings differently with every crack. It could also be due to the handheld antenna having a higher power than the lab-based system, due to the lower strength signal sent or the wider range of data collection locations this system was designed for. The scale of these values is no more than double that of Test 2a. The tag, when used in a different location with data collected from a different reader, still performed as intended. As the crack volume increased, so did the total damage index.

At the last three damage stages the damage index changing very little. This is believed to be because the most change in the damage index happened at initial crack development. The index then tapered off, even as the crack got larger. The range of this tag was not tested past a maximum crack volume of 0.0072 in^3^.

## 5. Conclusions

The RFID-based crack sensor was successfully used to monitor the increasing crack volume on UHPC. The crack volume range of this sensor has been confirmed to be from 0 to 0.0072 in^3^. This small range is due to the nature of the small cracks that UHPC develops. This low-cost commercial tags’ performance was validated in lab-based experiments with two different types of UHPC, with and without metal fibers, and two different systems (handheld and lab-based). The larger the total damage index, the larger the change in the volume of the crack since initial measurement. This development of a low cost crack sensing system has great potential for the monitoring of new material, UHPC, where retrofitting has been done. This can improve the quality control of the retrofitting process and help inspectors expedite their work. Further work to validate the performance of the system in the field is underway.

## Figures and Tables

**Table 1:** UHPC mix composition.

Components	Amount (lb/yd^3^)	Amount (kg/m^3^)
Premix Power	3700	2200
Water	219	130
Premia 150	30.0	18.0
Optima 100	20.0	12.0
Turbocast 650A	39.0	23.0
Steel Fibers (2%)	263	156

**Table 2:** Total damage index vs. crack volume: Test 2a.

Damage Stage	Crack Volume (in^3^)	Crack Volume (m^3^)	Total Damage Index (%)
1	0.00137	2.25 $\times 10^{-8}$	0.00990
2	0.00252	4.13 $\times 10^{-8}$	0.568
3	0.00303	4.97 $\times 10^{-8}$	1.29

**Table 3:** Total damage index vs. crack volume: Test 2b.

Damage Stage	Crack Volume (in^3^)	Crack Volume (m^3^)	Total Damage Index (%)
1	0.0000119	1.95 $\times \;10^{-10}$	0.177
2	0.00200	3.28 $\times \;10^{-8}$	1.06
3	0.00388	6.36 $\times \;10^{-8}$	1.18
4	0.00720	1.18 $\times \;10^{-8}$	1.25

**Table 4:** Total damage index vs. crack volume: Test 3.

Damage Stage	Crack Volume (in^3^)	Crack Volume (m^3^)	Total Damage Index (%)
1	0.0000194	3.18 $\times \;10^{-10}$	0.60
2	0.000670	1.10 $\times \;10^{-8}$	1.88
3	0.00137	2.25 $\times \;10^{-8}$	2.68
4	0.00253	4.15 $\times \;10^{-8}$	2.63
5	0.00303	4.97 $\times \;10^{-8}$	2.75
